# The Role of Cardiac Magnetic Resonance in mRNA COVID-19 Vaccine-Related Myopericarditis: An Evolutive Case Series

**DOI:** 10.3390/jcdd11090259

**Published:** 2024-08-25

**Authors:** Gisela Feltes, Violeta Sánchez Sánchez, Esther Pérez-David, José Luis Moreno-Hurtrez, Juan Delgado Jiménez, Iván J. Núñez-Gil

**Affiliations:** 1 Hospital Universitario de Torrejón, 28850 Madrid, Spain; feltesgg@vithas.es; 2 Hospital Universitario Vithas Arturo Soria, 28043 Madrid, Spain; sanchezsv@vithas.es (V.S.S.); delgadoj@vithas.es (J.D.J.); 3 Faculty of Biomedical and Health Sciences, Universidad Europea de Madrid, Villaviciosa de Odón, 28670 Madrid, Spain; eperezdavid@gmail.com; 4 Hospital Universitario 12 de Octubre, 28041 Madrid, Spain; 5 Hospital Universitario La Paz, 28046 Madrid, Spain; 6 Hospital Universitario Moncloa, 28027 Madrid, Spain; hurtrezj@vithas.es; 7 Hospital Clinico San Carlos, 28040 Madrid, Spain

**Keywords:** cardiac magnetic resonance, myocarditis, COVID-19, vaccines, case series

## Abstract

Numerous cases of myocarditis related to mRNA vaccines for COVID-19 have recently been described, usually in young men. Long-term evolutive cardiac magnetic resonance imaging (CMR) data are lacking. We describe four consecutive cases of COVID-19 vaccine-induced myocarditis. The pathological findings of cardiac magnetic resonance confirmed the diagnosis in the acute phase, showing edema, as well as pericardial enhancement, with light pericardial effusion and late gadolinium enhancement (LGE), predominantly in the inferolateral wall. These cases highlight the unique value of cardiac magnetic resonance in patients with suspected myocarditis induced by COVID-19 RNAm vaccines as a tool to confirm the diagnosis, avoiding other invasive techniques, as well as for the long-term follow-up of patients. Our iterative CMR imaging demonstrated frequent long-term LGE persistence.

## 1. Introduction

Numerous cases of myocarditis and pericarditis have recently been reported worldwide after the use of different mRNA vaccines for COVID-19, occurring more frequently in young men and after the administration of the second dose [1].

For the diagnosis, in addition to the clinical presentation of precordial pain, which is usually oppressive and sometimes exhibits pericardial characteristics, enzymatic elevation and abnormal electrocardiogram (EKG) findings are mandatory. On the EKG, repolarization alterations may sometimes be seen, which, when combined with the other abnormalities, can lead to the request for coronary angiography.

A recent systematic review showed the incidence of numerous new-onset rheumatic immune-mediated inflammatory diseases, including myocarditis, following COVID-19 vaccinations. Molecular mimicry is the theory with the strongest support for the occurrence of these adverse reactions after the administration of the vaccine. The adjuvant included in the vaccine probably shares structural similarities with self-antigens. Other studies suggest that the cause of these occurrences is due to the anti-SARS-CoV-2 spike antibodies or the SARS-CoV-2 recognition of T-cells that trigger prolonged immune-mediated inflammation [2].

Current evidence shows that the risk of myopericarditis from vaccination is lower than from COVID-19 infection, highlighting the benefits of vaccination. As previously noted, the mechanisms associated with the onset of this complication are not yet clearly defined, and more epidemiological data, as well as clinical and non-clinical research, are needed. Long-term pharmacovigilance and investigations are still being conducted by public entities and academic groups to determine the risk factors, clinical course, and outcomes associated with these patients [3].

The purpose of this series is to highlight the importance of a non-invasive test, such as cardiac magnetic resonance imaging (CMR), to confirm or rule out the diagnosis and to confirm its value for proper long term follow-up.

## 2. Materials and Methods

This is a single-center, observational (case-series) study of COVID-19 vaccine-induced myopericarditis in four patients. We retrospectively identified patients who were admitted to our Department of Cardiology between July 2021 and January 2022 with symptoms of myopericarditis and a history of COVID-19 vaccination. Physical examination, laboratory data, echocardiography results, and cardiac magnetic resonance imaging were considered for further analysis.

## 3. Case Presentation

### 3.1. Case 1

**Figure 1:**
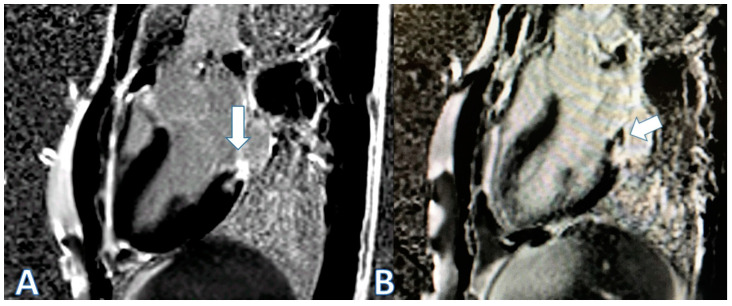
Cardiac MRI images. (**A**) Three-chamber view. Enhancement of both pericardial leaves and focal subepicardial LGE on basal inferolateral, lateral, and inferior walls (white arrow), with preserved biventricular function. (**B**) Three-chamber view, 3 months: persistence of focal LGE on basal inferolateral wall (white arrow).

We pose the case of a 20-year-old male who presented at the emergency room (ER) with oppressive precordial pain that worsened when the patient was lying down, but was not accompanied by fever or other symptoms. His only notable medical history was receiving the mRNA-1273 (Moderna, Cambridge, MA, USA) vaccine three days prior. He displayed elevated cardiac markers (peak TnT 181 ng/L [normal value < 14 ng/L]; ckMB 12 ng/mL [normal range: 0.0–3.6 ng/mL]); and elevated CRP (C reactive protein, peak 3.94 mg/dL [normal range: 0.0–0.5 mg/dL]), with slight ST elevation on the EKG, but a normal echocardiogram (ejection fraction, EF, 69%, normal E/e’ ratio). Cardiac magnetic resonance imaging (CMR) revealed late gadolinium enhancement (LGE) of both pericardial leaves and foci of late subepicardial enhancement on the basal inferolateral, lateral, and inferior walls, with preserved biventricular function (Figure 1A). He was hemodynamically stable, afebrile, without arrhythmias, and experienced only mild sporadic precordialgia. Treatment with NSAIDs (nonsteroidal anti-inflammatory drugs) was started, with a good response. He was discharged on the third day with ibuprofen and colchicine. A follow-up CMR at three months showed persistence of focal enhancement in LGE sequence on the inferolateral wall (Figure 1B). Another follow-up CMR at 9 months remained unchanged. At clinical follow-up, the patient remains asymptomatic.

### 3.2. Case 2

A 45-year-old male with a history of mild COVID-19 infection in March 2020 presented to the ER in July 2021 with chest pain of two hours’ duration. The pain was stabbing and radiated to the neck, with the onset four days after receiving the first dose of mRNA-1273 (Moderna, Cambridge, MA, USA). Elevated cardiac markers were noted (peak TnT 353 ng/L, ckMB 20.5 ng/mL), as well as elevated CRP (peak 3.63 mg/dL), leading to his admission to the intensive care unit (ICU). The EKG showed nonspecific repolarization abnormalities, and the echocardiogram was without segmental wall motion abnormalities (EF 60%, normal E/e´ ratio). Coronary heart disease was ruled out by coronary angiography. Cardiac magnetic resonance imaging (CMR) revealed mild late gadolinium enhancement (LGE) at the level of the pericardial leaves and a basal inferolateral subepicardial focus, consistent with myopericarditis. Treatment with NSAIDs and colchicine was administered. He remained asymptomatic, with normalization of cardiac markers, and was discharged without complications. At a 3-month follow-up, he remained asymptomatic, and control CMR showed the disappearance of LGE.

### 3.3. Case 3

**Figure 2:**
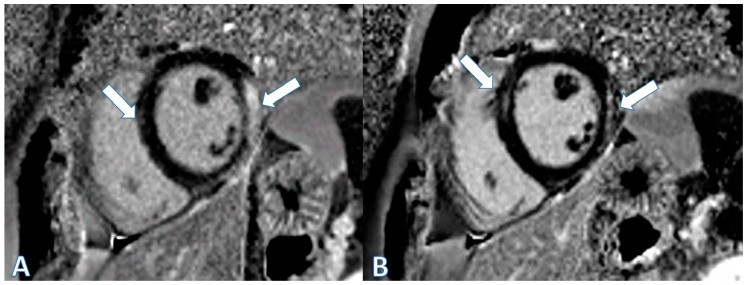
Cardiac MRI images. (**A**) Short-axis projection. Late enhancement sequence: subepicardial uptake affecting approximately 50% of the myocardial thickness in the lower-middle and inferolateral segments and the middle anteroseptal segment (arrows). (**B**). Short-axis projection. Follow-up at 2 years: persistence of LGE in the same locations, but with reduced extension (arrows).

The third case is a 34-year-old male with no previous pathological history who went to the ER after experiencing two episodes of precordial oppression radiating to the left arm, accompanied by difficulty swallowing and neck pain, that subsided spontaneously within 90 min and were not associated with exertion. He reported receiving the first dose of BNT162b2 mRNA (Pfizer-BioNTech, New York, NY, USA) 15 days prior. Admission to the ICU was determined due to an increase in cardiac biomarkers (TnT 286 ng/L; ckMB 12.1 ng/mL) and elevated CRP (peak 9.8 mg/dL). The EKG showed minimal repolarization changes in the lower leads, and the echocardiogram revealed no relevant findings (EF 60%, normal E/e´ ratio). CMR revealed edema and LGE in the lower middle, inferolateral, anterolateral and anteroseptal segments, compatible with acute myocarditis, with normal biventricular systolic function (Figure 2A). The patient had a good evolution, with controlled pain managed by NSAIDs and declining biomarkers since admission. He did not present signs or symptoms of heart failure and was discharged on day three with ibuprofen and colchicine. At the 6-month follow-up, he remained asymptomatic. Control CMR at 6 months showed the persistence of focal late enhancement in the inferolateral and anterolateral walls. Another control CMR was performed at 2 years, showing persistence of LGE in the same locations, albeit with reduced extent compared to that noted in the initial study (Figure 2B).

### 3.4. Case 4

**Figure 3:**
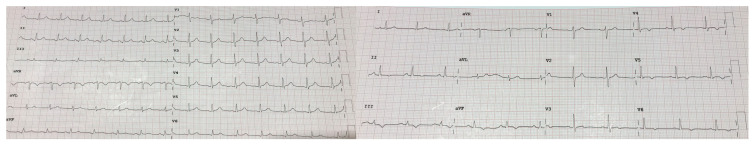
EKG at admission (**left**) and at discharge (**right**). Notice the appearance of negative T waves in the inferior and lateral leads.

**Figure 4:**
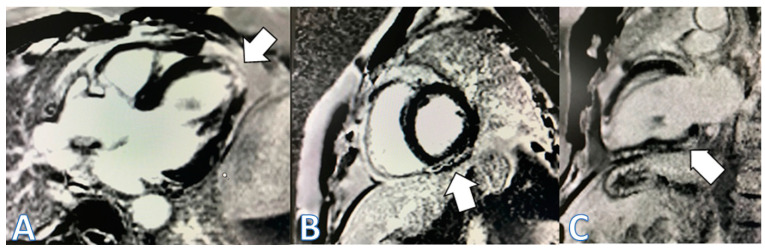
Cardiac MRI images. Left to right, (**A**) four-chamber view; (**B**) short-axis view; (**C**) two-chamber view. Late enhancement: intramyocardial and subepicardial patched foci in the anterolateral wall, basal-mid inferolateral and basal inferior segment, and intramyocardial foci at the mid-inferoseptal and apex segments (white arrows).

**Figure 5:**
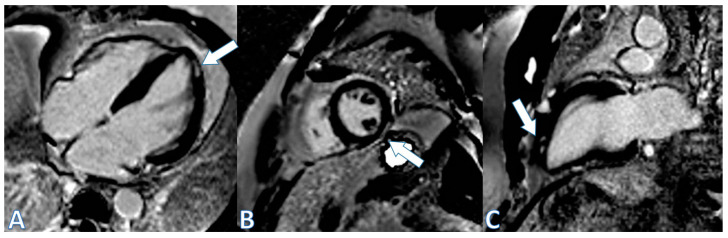
Cardiac MRI images. Left to right, (**A**) four-chamber view; (**B**) short-axis view; (**C**) two-chamber view. Follow-up at 9 months. Late enhancement: intramyocardial and subepicardial patched foci in similar locations, with reduced extension (white arrows) and disappearance at basal inferior segment.

The fourth case, a 48-year-old patient, received the third dose of the mRNA-1273 vaccine (Moderna, Cambridge, MA, USA) and presented with flu-like symptoms in the ensuing hours. Three days later, in the hours before admission, he experienced oppressive epigastric pain that did not radiate and was not accompanied by sweating or nausea. The pain worsened when the patient was lying down and improved partially when he was sitting, without being affected by deep inspiration. He went to the ER, where elevation in troponin up to 400 ng/L and CRP (peak 4.4 mg/dL) were detected, without changes in the EKG (Figure 3, left).The echocardiogram showed hypokinesia in the mid-distal inferolateral wall, a preserved LVEF of 57%, a normal E/e´ ratio, and no pericardial effusion. Coronary angiography was performed, showing normal results. CMR confirmed the diagnosis of myopericarditis. T2 STIR showed patchy hyperintense areas, suggestive of edema in the inferolateral and lateral walls, the apex of the LV, and the inferior basal segment. Intramyocardial and subepicardial patchy foci of LGE were observed in the anterolateral wall, basal-mid inferolateral, and basal inferior segments, with intramyocardial foci in the mid-inferoseptal and apex segments (Figure 4). He was treated with NSAIDs and discharged asymptomatic with ibuprofen and colchicine. Notably, negative T waves were observed in the inferior and lateral leads on the EKG at discharge (Figure 3 right). A control CMR was performed at the 9-month follow-up, showing the persistence of some intramyocardial and subepicardial foci at the inferolateral, anterolateral, and inferoseptal walls, which were smaller than those noted in the previous study, along with the disappearance of part of the LGE in other locations (Figure 5).

## 4. Discussion

Myocarditis and pericarditis after COVID-19 vaccination is a rare condition in which most patients who develop symptoms do so within a week of the administration, usually after receiving an mRNA vaccine (Pfizer or Moderna). Long-term follow-up data of hospitalized patients and the long-term consequences of this disease, confirmed by CMR, are not yet available.

We present four cases of myocarditis seen in the emergency room of our center (see Table 1). All patients were male, with an average age of 36 years (range 20–48 years), and with no history of cardiovascular disease. The average time from vaccination (Moderna in three cases and Pfizer in one case) to the onset of symptoms was six days. COVID-19 infection was ruled out in all patients.

All patients showed abnormal EKGs, with slight and non-specific changes in repolarization, and in some cases, slight ST segment elevation, leading to coronary angiography in two patients, which resulted in normal findings. Troponin T was elevated in all patients, with an average of 291 ng/L (normal value < 14 ng/L). Inflammatory blood markers were also elevated. The echocardiogram was normal, except in one patient, where inferolateral hypokinesia was observed. Diastolic function was normal in all cases. CMR was performed on all patients, which was crucial for confirming the diagnosis. In the STIR sequences, edema was observed in half of the cases. In the LGE sequences, involvement at the level of the pericardial leaves was demonstrated in two patients. Enhancement at the myocardial level was consistent in the inferolateral wall and was also observed in the lateral, inferior, and anterolateral walls in three patients, always subepicardial and intramyocardial. Mild pericardial effusion was observed in one patient. All cases showed a very good evolution, with symptom remission during admission, managed with NSAIDs, and discharge after an average of 3 days. It is interesting to mention that all patients were also treated with colchicine, some during hospitalization and others at discharge, for three months to prevent recurrences. This decision was based on the European guidelines on pericarditis, since there was no evidence in this context. Current studies support the use of colchicine in COVID-19 infection as an anti-inflammatory agent to reduce symptoms and hospitalization duration [4], oxygen requirements, and mortality [5]. Its utility was also described in the treatment of vaccine-induced myocarditis [6] and to prevent recurrences when a second dose of the vaccine was necessary [7].

Magnetic resonance imaging has proven to be a safe and effective technique for the diagnosis of patients in whom there is a high suspicion of myocarditis due to characteristic pain, elevation of cardiac enzymes and inflammatory markers, and mild abnormalities in EKG and echocardiogram data [8]. It can also help to avoid the use of invasive techniques, such as coronary angiography, in this type of patients. A recent study compared the CMR findings in classical myocarditis and in patients with COVID-19 vaccine-associated myocarditis. The results showed similar CMR findings and short-term outcomes, although the latter was associated with milder abnormalities and more pericardial involvement [9]. Another interesting study compared three groups of patients evaluated using CMR: those exhibiting myocarditis following vaccination, those displaying myocarditis due to COVID-19 infection, and those presenting with myocarditis due to other causes. The results showed that patients with vaccine-associated myocarditis had a higher left ventricular ejection fraction and less extensive LGE, with the subepicardial basal inferolateral wall as the most frequent location in all cases. The short-term follow up showed no adverse events in the vaccine-associated group [10].

Despite the unclear mechanisms for the development of myocarditis, several hypotheses have been suggested, including molecular mimicry between the spike protein of severe acute respiratory syndrome coronavirus-2 (SARS-CoV-2) and self-antigens, triggering of preexisting dysregulated immune pathways, immune response to mRNA, activation of immunologic pathways, and dysregulated cytokine expression [11]. However, despite this risk, some studies, like the international registry HOPE 2 (Health Outcome Predictive Evaluation for COVID-19), which recruited more than 9000 COVID-19 patients, suggested that vaccination was an independent protective factor for all-cause death, at least in patients with a previous heart condition [12].

A retrospective observational study suggested that despite the temporal association between an exposure to vaccine and myocarditis, it is always necessary to consider other potential causes, such as COVID-19 infection [13]. In our cases, however, COVID-19 infection was ruled out in all of them.

According to European and American guidelines for myocarditis [14,15], it is recommended to resume physical activity 3–6 months after the onset of symptoms due to the risk of arrhythmias, although there is no conclusive evidence in this context. Notably, these cases related to COVID-19 vaccination are milder and generally have a benign evolution compared to classic cases of myocarditis [16]. It is interesting to note that in one of our cases, complete resolution was observed in LGE sequences at 3 months, with persistence of LGE, to a lesser extent, in the other three, even 2 years later. The importance of late gadolinium enhancement is demonstrated in a study of 670 patients with viral myocarditis, followed up for almost 5 years, which demonstrated that LGE significantly modified the risk of future adverse cardiovascular outcomes, revealing that patients exhibiting mid-wall and septal LGE involvement showed a higher risk for MACE (major adverse cardiac events) [17].

The implications of persistent LGE in patients suffering from post-vaccination myopericarditis are still unknown, as is the time until its disappearance. A cardiac MRI follow up study showed persistence of minimal LGE in 48% of the patients after a median imaging follow-up of 214 days [18]. Therefore, magnetic resonance imaging would be a good non-invasive diagnostic method for monitoring the evolution in these patients and for authorizing the resumption of physical activity with certain guarantees, although more studies are needed to confirm these findings.

## 5. Conclusions

In conclusion, cardiac magnetic resonance imaging is a practical and non-invasive tool for the diagnosis and monitoring of patients with myocarditis induced by mRNA vaccines for COVID-19, potentially avoiding other invasive techniques. The present findings demonstrated that long-term late gadolinium enhancement persistence is common in this setting, despite an early and complete clinical recovery. The implications of these findings remain to be determined.

## Figures and Tables

**Table 1:** Demographics, diagnosis, and clinical outcomes of the patients following mRNA COVID-19 vaccination.

	PATIENT 1	PATIENT 2	PATIENT 3	PATIENT 4
Age (years old)	20	45	34	48
Sex	Male	Male	Male	Male
Previous cardiac disease	No	No	No	No
Symptoms onset after vaccination (days)	3	4	15	3
Vaccine type	mRNA-1273 (Moderna)	mRNA-1273 (Moderna)	BNT162b2 mRNA (Pfizer-BioNTech)	mRNA-1273 (Moderna)
Clinical characteristics	oppressive precordial pain	stabbing chest pain radiated to the neck	precordial oppression radiating to the left arm	oppressive epigastric pain
Troponin peak levels (ng/L)	181	353	286	400
CRP peak levels (mg/dL)	3.94	3.63	9.8	4.4
Coronary angiography	No	Yes	No	Yes
Treatment	NSAIDs and colchicine	NSAIDs and colchicine	NSAIDs and colchicine	NSAIDs and colchicine
Outcome	discharged on the 3rd day	discharged on the 3rd day	discharged on the 3rd day	discharged on the 3rd day
CMR at hospitalization	LGE of both pericardial leaves and foci of late subepicardial enhancement on the basal inferolateral, lateral, and inferior walls	LGE at the level of the pericardial leaves and a basal inferolateral subepicardial focus	Edema and LGE in the lower-middle, inferolateral, and anterolateral segments	Edema and LGE at the anterolateral, basal-mid inferolateral and basal inferior segments, and intramyocardial in the mid-inferoseptal and apex segments
Control CMR at 3 months	Persistence of LGE on the inferolateral wall	Disappearance of LGE	-	-
Control CMR at 6–9 months	Persistence of LGE on the inferolateral wall	-	LGE in the inferolateral and anterolateral walls	LGE intramyocardial and subepicardial at inferolateral, anterolateral, and inferoseptal walls (reduced extent)
Control CMR at 2 years	-	-	LGE in the inferolateral and anterolateral walls (reduced extent)	-

CRP: C reactive protein; CMR: cardiac magnetic resonance imaging; NSAIDs: nonsteroidal anti-inflammatory drugs; LGE: late gadolinium enhancement.

## Data Availability

Upon a reasonable email request to the corresponding author, the data presented in this study will be provided.
